# Neuroprotective Properties of Food-Borne Polyphenols in Neurodegenerative Diseases

**DOI:** 10.3390/antiox10111810

**Published:** 2021-11-15

**Authors:** Rui F. M. Silva, Lea Pogačnik

**Affiliations:** 1 Research Institute for Medicines (iMed.ULisboa) and Department of Biochemistry and Human Biology (DBBH), Faculty of Pharmacy, Universidade de Lisboa, 1649-003 Lisbon, Portugal; 2 Department of Food Science and Technology, Biotechnical Faculty, University of Ljubljana, 1000 Ljubljana, Slovenia; lea.pogacnik@bf.uni-lj.si

Fruits and vegetables are the richest source of polyphenols in the regular human diet. These substances belong to plants’ secondary metabolites and can have several roles, such as being metabolic intermediates, reproductive attractants, and protective agents. Most of these molecules possess a high antioxidant capacity, as well as several other important activities that can affect human health, among which anti-inflammatory properties and the potential ability to modulate different cell signaling pathways seem to be the most important. 

Taking into account these significant properties of polyphenols, together with their abundance in various food products that are a part of a healthy human diet, a wide range of different approaches, both in vitro and in vivo, address their potential role in the prevention and treatment of different pathological conditions associated with oxidative stress and/or inflammation.

The significance of food-borne polyphenols on human health has considerably increased the number of studies dedicated to their various proposed actions in many different pathologies such as cancer, and cardiovascular and neurodegenerative diseases, which has also resulted in a growing number of clinical trials on the acute or chronic use of dietary polyphenols. Studies in the field of cancer and neurodegenerative disorders are particularly important since an effective treatment is still not available. It was shown that food-borne polyphenols can be used either as protective/prophylactic molecules or as therapeutic substances. They can be consumed as part of a natural polyphenol-enriched diet, with the use of food supplements or as pharmaceutical drugs/nutraceuticals. 

In this Special Issue of *Antioxidants*, several research papers and two reviews explore the chemical properties of naturally occurring polyphenols and some new possibilities for the therapeutic and/or prophylactic roles of these molecules in neurodegeneration and neurodegenerative diseases.

Ribeiro et al. [1] proposed the use of a new enzymatic biosensor to determine the antioxidant activity of commercially available teas, as well as the level of ascorbic acid in effervescent products, which can also be used to detect the real level of antioxidants in samples and depict the validity of “antioxidant” labeling in the product information. Using flavone derivatives, Sakalauskas et al. [2] further explored the potential of polyphenols to prevent amyloid aggregation, an important hallmark of Alzheimer’s disease (AD) as well as other amyloid-related disorders. They demonstrated that after oxidation, flavones, particularly oxidized 6,2′,3′-trihydroxyflavone, not only keep but even increase their anti-amyloid properties, which might be a relevant addition to the discussion relating to whether, after physiologic metabolization, polyphenol derivatives still keep their beneficial properties. 

Continuing with the study of the antioxidant properties of polyphenol-rich vegetables, Jug et al. [3] evaluated extracts of Japanese knotweed rhizomes produced by several extraction solvents. Knotweed is an invasive botanical species, and it is important to find novel economically viable applications for this destructive botanical species. Using size exclusion–high-performance liquid chromatography (SEC-HPLC)-UV and reversed-phase HPLC-UV coupled with multistage mass spectrometry to fractionate the extracts, they identified (−)-epicatechin as a potent and stable antioxidant.

The well-proven antioxidant properties of polyphenols can be explored in the prevention of or reduction in oxidative stress-induced injury. Accordingly, the results from Bobadilla et al. continue to reinforce the potential of natural food polyphenols to reduce neuronal demise by several mechanisms, as in the case of aluminum maltolate neurotoxicity, where several commercially available natural food supplements decreased neuronal cell death in vitro by reducing ROS levels and caspase-3 activity, and also increased the antioxidant enzyme activity in mice, preventing the formation of lipid peroxidation products in the brain [4]. 

The multiple targets for polyphenols and their beneficial effects on several human chronic diseases were reviewed by Bucciantini et al. [5]. These authors explore, in particular, the actions from polyphenols present in extra virgin olive oil, expanding from the antioxidant to the anti-inflammatory properties. They advance to the use of olive oil polyphenols in human chronic diseases that involve inflammation due to their inhibitory effects on oxidative stress-induced signaling pathways and minimal secondary effects.

The second review from this Special Issue reinforces polyphenols’ multiple mechanisms of action, focusing on amyotrophic lateral sclerosis (ALS) and frontotemporal dementia (FTD), two of the neurodegenerative disorders still without effective treatments or cures. For that, Novak et al. [6] analyze the current therapeutical options for ALS and FTD in parallel with several of the more prominent polyphenols such as resveratrol, curcumin and green tea catechins, emphasizing the therapeutic potential of polyphenols.

In fact, according to the results from Zheng et al. [7], resveratrol might also influence the clearance of beta-amyloid peptide-42 (Aβ42), related to AD neurodegeneration, by modulating the insulin-degrading enzyme that also has a strong ability to degrade Aβ4. 

Moving to in vivo studies, a crucial pre-clinical step, the findings from Hong et al. [8] show very important amelioration effects on cognitive and memory functions in models of neurodegeneration. Interestingly, ampelopsin A from Vitis vinifera was shown to have neuroprotective properties, increasing both cognitive and memory functions by, in part, elevating the BDNF/CREB-related signaling, in a mice model as well as in hippocampal brain slices (CA3-CA1 synapses), where neurodegeneration was induced by scopolamine. 

Finally, in a pilot study performed in older human volunteers with memory complaints, but not AD, Robinson at al. [9] evaluated the effects of the administration of a whole coffee cherry extract nutraceutical, rich in polyphenols, using MRI and determination of BDNF blood levels. In summary, they found significant improvements in cognition that may be related to the increase in exosomal BDNF. 

In conclusion, polyphenols seem to be effective molecules for preventive and therapeutic strategies in a wide range of pathological conditions. However, it will be important to take into account the possible issues raised by their dosage and toxicity and monitoring of their safe usage. 
